# The historical role of species from the Solanaceae plant family in genetic research

**DOI:** 10.1007/s00122-016-2804-1

**Published:** 2016-10-15

**Authors:** Christiane Gebhardt

**Affiliations:** MPI for Plant Breeding Research, Cologne, Germany

## Abstract

**Key message:**

**This article evaluates the main contributions of tomato, tobacco, petunia, potato, pepper and eggplant to classical and molecular plant genetics and genomics since the beginning of the twentieth century.**

**Abstract:**

Species from the Solanaceae family form integral parts of human civilizations as food sources and drugs since thousands of years, and, more recently, as ornamentals. Some Solanaceous species were subjects of classical and molecular genetic research over the last 100 years. The tomato was one of the principal models in twentieth century classical genetics and a pacemaker of genome analysis in plants including molecular linkage maps, positional cloning of disease resistance genes and quantitative trait loci (QTL). Besides that, tomato is the model for the genetics of fruit development and composition. Tobacco was the major model used to establish the principals and methods of plant somatic cell genetics including in vitro propagation of cells and tissues, totipotency of somatic cells, doubled haploid production and genetic transformation. Petunia was a model for elucidating the biochemical and genetic basis of flower color and development. The cultivated potato is the economically most important Solanaceous plant and ranks third after wheat and rice as one of the world’s great food crops. Potato is the model for studying the genetic basis of tuber development. Molecular genetics and genomics of potato, in particular association genetics, made valuable contributions to the genetic dissection of complex agronomic traits and the development of diagnostic markers for breeding applications. Pepper and eggplant are horticultural crops of worldwide relevance. Genetic and genomic research in pepper and eggplant mostly followed the tomato model. Comparative genome analysis of tomato, potato, pepper and eggplant contributed to the understanding of plant genome evolution.

## Introduction

The Solanaceae family comprises 3000–4000 species that are classified in approximately 90 genera. The family is highly diverse, includes perennial trees as well as herbaceous annual species and occupies a wide range of terrestrial habitats from deserts to rainforests (Knapp et al. 2004). Compared with the large size of the family, only few members of the Solanaceae attained importance in human civilizations as food sources [potato, tomato, pepper, eggplant, pepino, naranjilla, tamarillo (tree tomato)], ornamentals (petunia, *Datura*, some *Solanum* species, *Schizanthus*) and drugs (tobacco, *Atropa, Hyoscyamus, Mandragora*). After the re-discovery and confirmation of Gregor Mendel’s pioneering work (Mendel 1866) by Hugo de Vries and Karl Correns around the beginning of the twentieth century, species in seven genera of the Solanaceae became objects of genetic research as model plants and/or due to their importance as crops. Model plants, first in classical and then in cellular and molecular genetics, were primarily the cultivated tomato and its wild relatives (genus *Solanum,* former genus *Lycopersicon*), tobacco (genus *Nicotiana*) and petunia species (genus *Petunia*). The cultivated potatoes, the eggplant or aubergine (genus *Solanum*) and pepper species (genus *Capsicum*) are crop plants of worldwide importance. These species are less suitable as models for fundamental research, except tuber development in potato. Classical and molecular genetic research on potato, eggplant and pepper was, therefore, predominantly aimed at solving agricultural problems, for example, introducing disease resistance, and developing improved cultivars. *Datura innoxia* (genus *Datura*), *Hyoscyamus muticus* (genus *Hyoscyamus*) and *Physalis* species (genus *Physalis*) were not widely used in research but, nevertheless, contributed to certain aspects of genetics. Anthers of *Datura innoxia* were cultured in vitro in the earliest conclusive report on the regeneration of haploid embryos from pollen grains (Guha and Maheshwari 1966). This marked the beginning of the production of doubled haploid and, therefore, homozygous plants, which is now an important biotechnological application in breeding programs of all self-fertile crop species, because homozygous plants can be obtained in a single step and much shorter time, therefore, than by multiple generations of selfing. Leaf mesophyll protoplasts of anther culture-derived haploid *Hyoscyamus muticus* plants were the source of biochemical mutant cell lines that were obtained by total selection (Gebhardt et al. 1981), similar to the first biochemical mutants selected in *Neurospora crassa* (Beadle and Tatum 1941). The molecular basis and possible mechanism of the formation of the ‘Chinese lantern’ or inflated-calyx syndrome, a peculiar development of the sepals after fertilization in some Solanaceae genera, were studied in *Physalis* species (He and Saedler 2005, 2007).

The aim of this review was to highlight the historical roles and main contributions of tomato, tobacco, petunia, potato, pepper and eggplant in genetic research since the beginning of the twentieth century. The broadness of the subject required selectivity, which might have been influenced by the author’s own experience and preferences. The undoubtedly important contributions of cytogenetics were neglected as well as the numerous studies on gene function based on transgenic plants. Particular attention was paid to the identification and citation of the early original literature on genetic research in Solanaceous species. This might appear outdated in view of today’s era of sequenced genomes but, nevertheless, formed the humus, which nourished the research that led to the manifold biotechnological applications in plant breeding, such as in vitro multiplication, doubled haploid production, genetic engineering and marker-assisted breeding.

## Tomato

The nine tomato species in the former genus *Lycopersicon*, now included in the genus *Solanum* based on molecular taxonomy (https://solgenomics.net/about/solanum_nomenclature.pl), are native to Middle and South America. The cultivated tomato (*Solanum lycopersicum*, formerly *Lycopersicon esculentum*) was domesticated in pre-Colombian times in Mexico or Peru and was introduced into Europe shortly after the Spanish conquest of Mexico in 1521. The first descriptions of the ‘love apple’ appeared in European herbals around the middle of the sixteenth century (Daunay et al. 2008). From there, the tomato spread to all other continents and is today the second most important Solanaceous crop plant after potato. The cultivated tomato was, besides maize, the major plant model of classical genetics in the twentieth century, thanks to its self- and cross-compatibility, easy cultivation and large morphological diversity. The genetic linkage map of tomato was initiated in the first decade of the twentieth century by analyzing the Mendelian inheritance of morphological characters such as plant dwarfism and color, shape and surface structure of fruits and leaves (Craig 1907; Halsted 1905; Hedrick and Booth 1907; Price and Drinkard 1908). The discovery of genetic linkage between some of these characters formed the core of the classical tomato map (Jones 1917). Seven decades later, the map comprised nearly 320 morphological and isozyme loci (Mutschler et al. 1987). A significant portion of the morphological markers originated from more than 300 radiation-induced mutants, which were generated and described by Hans Stubbe and colleagues from the Institute for Genetics and Crop Plant Research in Gatersleben (former German Democratic Republic) in the 1950s and 1960s (Stubbe 1957, 1972). Several genes underlying morphological characters that were described and mapped many years ago by classical geneticists have now been molecularly identified by positional cloning, for example: the transcription factors *lateral suppressor* (*Ls*) and *blind* (*bl*) which control axillary meristem formation (Schmitz et al. 2002; Schumacher et al. 1999), the regulatory gene *ovate* (*o*) which controls fruit shape (Liu et al. 2002) and the MYB transcription factor *potato leaf* (*C*) which changes leaf shape (Busch et al. 2011). After the beginning of the twenty-first century, mutant collections of tomato were expanded by several thousand, providing new opportunities for the discovery and analysis of gene function (Menda et al. 2004; Saito et al. 2011).

Linkage mapping based on morphological and isozyme markers became obsolete about 30 years ago when DNA-based markers appeared on stage in the form of restriction fragment length polymorphisms (RFLPs) (Helentjaris et al. 1985). Whereas the construction of the classical tomato map took 80 years, hundreds of crosses and several generations of geneticists, the construction of a molecular linkage map with hundreds of RFLP markers was achieved within less than 10 years by two dozen researchers based on single F2 populations that descended from crosses between cultivated tomato and wild tomato species (Bernatzky and Tanksley 1986; Helentjaris et al. 1986; Tanksley et al. 1992). The tomato RFLP maps were, together with maize, the first molecular linkage maps generated in plants. RFLP linkage maps were the first genomic tool that made possible (i) the genetic dissection and mapping of quantitative trait loci (QTL), (ii) the cloning of genes with phenotypic effect but unknown sequence based on map position alone and (iii) the marker-assisted introgression of novel traits, for example, disease resistance, in breeding programs. DNA-based markers for most tomato diseases and fruit quality traits have been identified by linkage mapping in progeny of interspecific hybrids. Their application in commercial breeding programs has been recently reviewed (Foolad and Panthee 2012). The use of interspecific hybrids as parents was necessary because of the scarcity of intraspecific DNA variation in the cultivated tomato, which made detection by RFLPs tedious. Twenty years later, next-generation sequencing technologies have improved, tremendously, the efficiency of detecting DNA variation and allow now map construction in intraspecific populations based on single nucleotide polymorphism (SNP) markers (Shirasawa et al. 2010). High-density molecular linkage maps were instrumental for the construction of a physical map, by which the bits and pieces of assembled genomic sequences (scaffolds) were ordered and linked to the 12 chromosomes of the inbred cultivar ‘Heinz 1701’, the first sequenced tomato (The Tomato Genome Consortium 2012). Besides this reference genome of the cultivated tomato, the genomes of the wild species *S. pimpinellifolium* and *S. pennellii* were also sequenced, which are sources of valuable horticultural traits (Bolger et al. 2014; The Tomato Genome Consortium 2012).

The earliest example for map-based gene cloning in plants was the tomato *Pto* gene for resistance to *Pseudomonas syringae* pv tomato, which turned out to encode a serine–threonine kinase (Martin et al. 1993a, b). The first QTL mapping experiments used mainly RFLP and some isozyme markers. QTL for insect resistance (Nienhuis et al. 1987) and for important fruit quality traits such as soluble solids concentration (mainly sugars), fruit weight and acidity (Osborn et al. 1987; Paterson et al. 1988) were reported. Since then, numerous QTL mapping studies have been performed in various interspecific populations, reviewed by (Grandillo et al. 2013), see also Bernardo (2016) in this issue. QTL mapping was the starting point for the positional cloning of genes underlying QTL. The first example was a major QTL for fruit weight (*fw2.2*) on tomato chromosome 2, which turned out to be a gene of unknown function, possibly a regulator of cell division (Frary et al. 2000; Grandillo and Tanksley 1996). Particularly useful for QTL mapping and cloning was and still is a set of introgression lines of *S. pennellii* in *S. lycopersicum*, which was developed based on marker-assisted selection with RFLP markers (Eshed and Zamir 1995). This material led finally to the identification of an invertase gene that underlies a major QTL for fruit sugar content (Fridman et al. 2004).

Progeny of controlled crosses between two inbred parental lines was the material that dominated plant genetics in the last century. Since the beginning of the twenty-first century, the principles of population genetics, originally developed in human genetics, are increasingly applied to plant populations of individuals related by descent, which either occur in natural habitats or are the result of breeding. Such populations arise from crosses among multiple parents over multiple generations. Instead of two parental alleles per locus, which segregate in Mendelian ratios, multiple alleles are distributed in these populations in frequencies that are the result of evolutionary forces such as natural selection and random genetic drift, or selection by humans. Instead of the recombination frequency, the frequency of co-inheritance of specific alleles at pairs of linked loci over multiple meiotic generations is estimated (linkage disequilibrium, LD). The phenotypic and genotypic analysis of such populations allows the detection of association or LD between a quantitative or qualitative phenotypic trait and a specific allele of a DNA-based marker. Such marker–trait associations are highly useful for marker-assisted breeding as they are not restricted to a specific genetic background. Association mapping has its limitations in highly inbred crops like tomato, because the genetic resolution is low due to large LD. Nevertheless, first examples of QTL detection by association mapping were recently published (Ranc et al. 2012; Ruggieri et al. 2014; Sauvage et al. 2014). Multiparent advanced generation inter-cross (MAGIC) populations combine advantages of linkage mapping such as controlled crossings with advantages of association mapping such as segregation of multiple alleles and increased genetic resolution. A first MAGIC population was recently developed in tomato as a novel, alternative genetic resource for QTL mapping and cloning (Pascual et al. 2015).

Naturally, tomato is the prime model for studying the molecular basis of fruit development and composition. Spontaneous and induced mutations for fruit ripening, fruit shape, color and texture were described by classical geneticists in the twentieth century. At the end of the twentieth and beginning of the twenty-first century, many of the corresponding genes were cloned and functionally characterized, either by positional cloning or the candidate gene approach. This revealed the importance of ethylene signaling and particular transcriptional regulators in fruit ripening (reviewed by (Giovannoni 2007; Klee and Giovannoni 2011). The FLAVR SAVR™ tomato with prolonged shelf life due to antisense inhibition of polygalacturonase in the fruit was the first commercialized transgenic crop (Kramer and Redenbaugh 1994).

## Tobacco

In 1492, Christopher Columbus noticed smoking indigenous people in Mexico and Cuba. In the middle of the sixteenth century, several tobacco species, the first one probably *Nicotiana rustica*, were introduced into Portugal and France from the New World (Daunay et al. 2008). In the following centuries, tobacco conquered the world as a powerful drug in the form of cigarettes, cigars and pipes. In twentieth-century plant science, tobacco was the most widely used model for the development of somatic cell genetics including transformation technology and genetic engineering. The in vitro multiplication of plant cells and tissues, the production of doubled haploids and transgenic crops, which are important parts of today’s green biotechnology are all rooted, to large extent, in basic research performed with tobacco cell and tissue cultures, reviewed by (Sussex 2008). The ability of de-differentiated plant cells to grow indefinitely in vitro was conclusively demonstrated with tumor-like callus tissue derived from a hybrid between *N. glauca* and *N. langsdorffii* (White 1939b). The next step was the regeneration of roots, shoots and, finally, normal, fertile plants from undifferentiated tobacco cell cultures, which involved the discovery that the plant hormones auxin and cytokinin had to be added to the culture medium in the right concentration and ratio to induce the differentiation of roots or shoots (Skoog 1944; Skoog and Tsui 1948; White 1939a). The question then was whether single somatic plant cells are totipotent, that is whether they are able to develop by cell division and organ differentiation into a fully functional and fertile plant. The totipotency of somatic plant cells was shown using tobacco single cell cultures (Muir et al. 1954; Vasil and Hildebrandt 1965a, b) and, later, leaf mesophyll protoplasts (Nagata and Takebe 1971; Nakata and Tanaka 1968). All this was made possible by improvements of the culture media. The first fully defined plant tissue culture medium ‘MS’ was developed for tobacco cell cultures and is still widely used for the in vitro propagation of many plant species (Murashige and Skoog 1962). Another important development was the regeneration of haploid plants from pollen grains, which was achieved by in vitro culture of tobacco anthers (Bourgin and Nitsch 1967; Nitsch and Nitsch 1969; Vasil and Nitsch 1975). When it became possible to proceed in vitro from single cells to plants, plant cell cultures could be used like microbial cultures for the isolation of biochemical mutants, reviewed by (Negrutiu et al. 1984). The first such mutants were obtained using tobacco cells. Mutant cell lines were selected based on resistance to chemicals such as chlorate, 5’ bromo-deoxyuridine and methionine sulfoximine, which kill wild-type cells whereas mutant cells survive (Carlson 1970, 1973; Márton et al. 1982a, b; Müller and Grafe 1978). A milestone in plant science and biotechnology was the transformation of tobacco cells with T-DNA or Ti-plasmid carrying *Agrobacterium tumefaciens*, which resulted in the stable integration and inheritance of foreign genes (Chilton et al. 1977; De Block et al. 1984; Hernalsteens et al. 1980; Horsch et al. 1984). The simple *Agrobacterium*-mediated transformation of differentiated tissues such as leaf discs was demonstrated with tobacco, tomato and petunia (Horsch et al. 1985).

Tobacco species served as models to study the genetics and molecular basis of gametophytic self-incompatibility. During the first quarter of the twentieth century, extensive studies on the inheritance of self-incompatibility (self-sterility at that time) were performed with hybrids between *N. alata, N. forgetiana* and *N. langsdorffii* (Anderson 1924; East and Mangelsdorf 1925; East and Park 1917). Seven decades later, the first gene for gametophytic self-incompatibility was cloned from *N. alata,* which encoded a stylar glycoprotein (Anderson et al. 1986). The mechanism of self-incompatibility was subsequently elucidated by showing that this glycoprotein functions as an RNase that degrades pollen rRNA (McClure et al. 1989, 1990). Research on the host–pathogen system tobacco–*Tobacco Mosaic Virus* (TMV) marks the beginning of plant virology hundred years ago, reviewed by (Scholthof 2008). The dominant gene *N* from *N. glutinosa* that confers hypersensitive resistance to TMV (Holmes 1938) was finally cloned by transposon tagging in *N. tabacum* (Whitham et al. 1994). *N* was the first gene for virus resistance that was sequence-characterized and is the founder of a major class of plant resistance genes that are characterized by a Toll–interleukin receptor-like (TIR) domain, a nucleotide binding site (NBS) and a leucine-rich repeat (LRR) domain. More recently, *N. attenuata* became a productive model for elucidating the molecular basis of plant interactions with insect herbivores, reviewed by (Schuman and Baldwin 2016).

*Nicotiana benthamiana* is a tobacco species endemic to Australia. Since about twenty years, laboratory accessions of this species have become an important experimental system for the functional analysis of host–pathogen interactions, in particular virus interactions, as well as for studies on protein localization and protein–protein interactions, reviewed by (Goodin et al. 2008). This is based on the amenability of *N*. *benthamiana* to infection with most viruses, which allows the introduction and transient expression of foreign genes cloned in plant viral vectors in sense or antisense orientation (Donson et al. 1991; Kumagai et al. 1995). Another advantage of *N*. *benthamiana* is the ease by which it is transformed via infiltration of leaves with *Agrobacterium tumefaciens* (agroinfiltration), which enables high-throughput functional screens.

Genomics arrived late in tobacco compared to other crop species. The first linkage map was constructed with microsatellite markers (Bindler et al. 2006, 2011). At present, draft genome sequences are available of three varieties of *N. tabacum* (Sierro et al. 2014) and of *N. benthamiana* (Bombarely et al. 2012).

## Petunia

Petunias are one of the most popular annual bedding plants in Europe and the United States. South America is the home of the approximately 30 known species of the genus *Petunia*, which was established in 1803 by the French botanist Antoine-Laurant de Jussieu. The first wild *Petunia* species were cultivated in the botanical gardens of Berlin (1823) and Glasgow (1831) from seeds introduced from the southern half of South America (Gerats and Vandenbussche 2005; Krausch 2007; Sink 1984). The great diversity seen today in flower color, size and pattern as well as plant growth habit is the result of 170 years of breeding and selection, which adapted and continues to adapt this ornamental plant to the fancies of humans who love to decorate their windows, balconies and gardens with colorful flowering plants.

It is, therefore, conceivable that, after the re-discovery of Mendel’s work, petunia flower morphology was among the earliest heritable plant characters studied by means of controlled crosses. In the very first issue of the oldest British journal of genetics, Edith R. Saunders published a paper on the inheritance of ‘doubleness’ versus ‘singleness’ of petunia flowers (Saunders 1910). In the second half of the twentieth century, petunia, together with snapdragon (*Antirrhinum majus*), became the model for classical and molecular genetics of flower pigmentation by anthocyanins, a subclass of the flavonoids (Gerats and Vandenbussche 2005; Holton and Cornish 1995; Winkel-Shirley 2001). The genetic basis was the countless flower color variation of *Petunia hybrida* that was selected by breeders from progeny of interspecific hybrids or from spontaneous mutations. Since an early study on the inheritance of petunia flower colors in the context of understanding speciation (Mather and Edwardes 1943), more than thirty color mutants have been described and mapped to the seven petunia chromosomes (de Vlaming et al. 1984). Subsequently, many structural and regulatory genes in flavonoid biosynthesis were cloned and molecularly characterized from maize, snapdragon and petunia (Dooner et al. 1991; Gerats and Vandenbussche 2005). In the early days of gene cloning in plants, the first petunia genes were cloned based on sequence homology with the corresponding parsley gene. They encoded chalcone synthase, the key enzyme in flavonoid biosynthesis (Reif et al. 1985). The observation and genetic analysis of unstable alleles at flower pigmentation loci (Bianchi et al. 1978; Doodeman et al. 1984; Farcy and Cornu 1979) led finally to the discovery and cloning of the non-autonomous transposable element dTph1 (Gerats et al. 1990, 2013). Transposon tagging based on dTph1 and the autonomous element Act1, combined with twenty-first-century sequencing technology is now considered as a powerful tool for forward and reverse genetics in petunia. Thousands of insertion mutants were generated using the Act1/dTph1 transposable element system, which led to the identification of interesting novel genes (Van Houwelingen et al. 1998; Vandenbussche et al. 2016).

A hallmark in the history of transgenic plants was the genetic engineering of petunia flower color. The *A1* locus of maize encoding the enzyme dihydroquercetin 4-reductase (DQR) (O’Reilly et al. 1985; Schwarz-Sommer et al. 1987) is required for the biosynthesis of pelargonidin, which confers a brick-red color to flowers. Pelargonidin is produced, for example, in geranium flowers but not in petunia flowers. The maize *A1* gene was transferred by protoplast transformation into a pale pink flowering petunia mutant that accumulated the substrate of DQR, and transgenic plants with salmon red flowers were obtained (Meyer et al. 1987). The first field experiment with transgenic plants was conducted with 30,000 plants of one transgenic petunia line at the Max Planck Institute for Plant Breeding Research in the summer 1990. The planting was accompanied by protests against the release of transgenic plants (witnessed by the author). This experiment demonstrated that transgene expression and, thereby, flower color could be reduced or even silenced by methylation of the 35S promoter driving the expression of the transgene, which was correlated with developmental and environmental factors (Meyer et al. 1992). The genetic and molecular analysis of transgenic petunia plants with modified flower color by these and other researchers (Napoli et al. 1990; Van der Krol et al. 1990) made important contributions to the discovery of co-suppression and gene silencing in plants.

In addition to *Arabidopsis thaliana* and *Antirrhinum majus* (Schwarz-Sommer et al. 1990), *Petunia hybrida* is a model for the molecular genetics of flower development. Several homeotic and non-homeotic flower mutants have been described (de Vlaming et al. 1984; Saunders 1910). The identification and functional analysis of the underlying genes showed similarities as well as differences between the transcriptional networks that control flower development and organ identity in plant species from different genera (Heijmans et al. 2012; Mach 2012; Van der Krol and Chua 1993).

Compared with tomato, potato and pepper, genomic tools like molecular linkage maps and genome sequences are less developed in petunia, probably due to the large genome size and severely reduced recombination frequencies in progeny of *Petunia hybrida* (Bossolini et al. 2011; Strommer et al. 2002). A draft genome sequence is currently under construction (Vandenbussche et al. 2016).

## Potato

All of the approximately 180 tuber-bearing *Solanum* species are indigenous to Latin America. They occur in a wide range of habitats from Mexico in the North to Chile in the South. Archaeological evidence from the Peruvian cost indicated that potato cultivation dates back to approximately 9000 years. After the Spanish conquest of the Inca state in 1532–1536, the first potatoes were introduced into Europe, most likely via the Canary Islands, where the ships anchored on their way from America to Europe (Hawkes 1990; Ríos et al. 2007). In the following four centuries, potato cultivation slowly spread throughout Europe and, from there, to the rest of the world. Today, potato is, in terms of production, the world’s third food crop after wheat and rice. The seminal paper on potato genetics was published by Redcliffe N. Salaman in the first issue of the Journal of Genetics (Salaman 1910). Genetic analysis in potato proved difficult right from the beginning due to low fertility and problems in scoring morphological characters accurately as Mendelian factors. Nevertheless, Salaman reported Mendelian inheritance of pollen sterility, tuber shape, eye depth and color, and resistance to *Phytophthora infestans* in a wild potato species (named at that time *S. etuberosum* but was probably *S. demissum*). Years later, the tetraploid nature and tetrasomic inheritance of the European cultivated potato were established (Cadman 1942; Rybin 1930; Smith 1927), which leads to Mendelian segregation ratios for only two of twelve possible allelic states of heterozygous parents (*Aaaa* x *aaaa* and *Aaaa* x *Aaaa*). Potato cultivars were and still are non-inbred and, therefore, highly heterozygous. They are generated by intercrossing non-inbred parents, and heterozygous genotypes are maintained by vegetative propagation of the tubers. Classical genetic analysis in the cultivated potato remained difficult and was, therefore, limited. Exceptional were the studies of Aksel P. Lunden on the inheritance of color in tubers and flowers (Lunden 1960) and of G. Cockerham on the inheritance of resistance to the potato viruses X and Y (Cockerham 1970). One of the seven genes for resistance to *Potato Virus X* (PVX) analyzed by Cockerham (*Rx*_*adg*_) was the first potato gene that was identified 30 years later by positional cloning (Bendahmane et al. 1999).

The reduction of the ploidy level from tetraploid to diploid greatly facilitated potato genetics, reviewed in (Rokka 2009). This was achieved either by the parthenogenetic development of 2n female gametes after pollination with certain genotypes of the closely related species *S. phureja* (Hougas et al. 1958) or by anther culture of 4n cultivars and regeneration of 2n plants from male gametes (Dunwell and Sunderland 1973). The diploid potatoes obtained by these methods were sexually self-incompatible though. Thus, the genetics of diploid, heterozygous, self-incompatible potatoes is equivalent to human genetics. The discovery of the *Sli* (*S locus inhibitor*) gene from the wild potato species *S. chacoense*, which confers self-compatibility (Hosaka and Hanneman 1998), might mark the beginning of a new era in potato genetics and breeding, where pure diploid lines can be generated and used in hybrid breeding (Lindhout et al. 2011). The first potato linkage maps were constructed using RFLP markers and progeny of interspecific (Bonierbale et al. 1988) and intraspecific crosses (Gebhardt et al. 1989, 1991) between heterozygous, diploid parents. Twenty years later, the ultimate map, that is the genomic sequence corresponding to the twelve potato chromosomes, was assembled from DNA sequences of an exceptional, because doubled monoploid and, therefore, homozygous diploid genotype of *S. phureja*, a diploid potato species cultivated in Colombia and Peru (PGSC 2011; Sharma et al. 2013).

The more closely related two species are, the greater is the sequence similarity between their genomes. As RFLP markers were detected by a DNA–DNA hybridization assay (Southern blot analysis) that depends on sequence similarity, RFLP markers originated from DNA of one species could be used for linkage map construction in other, related species. Prior to the era of whole genome sequences, the construction of linkage maps in two species with the same set of RFLP markers allowed for the first time the comparison of genome structure between sexually incompatible species and, thereby, the deduction of models for genome evolution and speciation. This concept of genome synteny was tested for the first time in plants by constructing a potato linkage map with tomato RFLP markers of known position on the tomato linkage map (Bonierbale et al. 1988). This showed that the order of the markers was largely the same in potato and tomato, except five inversions of chromosome arms, as evidenced by inverted marker order. Even between species as distantly related as potato and the model plant *Arabidopsis thaliana*, genome fragments with conserved marker order and, therefore, conserved genome structure (syntenic blocks) could be detected (Gebhardt et al. 2003). Today, the comparison of whole genome sequences confirms and refines the syntenic relationships between Solanaceous species, for example (Doganlar et al. 2002a; Hirakawa et al. 2014).

RFLP linkage maps were the starting point for mapping in experimental, diploid populations, first, major genes for pathogen resistance (Barone et al. 1990; Leonards-Schippers et al. 1992; Ritter et al. 1991), reviewed by (Simko et al. 2007), and, after that, resistance QTL (Bonierbale et al. 1994; Leonards-Schippers et al. 1994; Simko et al. 2007) and a range of agronomically important, complex tuber characters such as starch content, chip color, yield, dormancy and susceptibility to bruising (Douches and Freyre 1994; Freyre et al. 1994), reviewed by (Gebhardt et al. 2014). Molecular cloning of the first two plant resistance genes of the NBS-LRR type, *N* and *RPS2* from tobacco and Arabidopsis, respectively, (Bent et al. 1994; Mindrinos et al. 1994; Whitham et al. 1994) allowed the identification of conserved sequence motifs, which were the basis for the amplification by PCR (polymerase chain reaction), the cloning and RFLP mapping of ‘resistance gene like’ (RGL) sequences in potato (Leister et al. 1996). Co-segregation of one specific RGL with the *Gro1* locus for resistance to the root cyst nematode *Globodera rostochiensis* was instrumental for the cloning of the nematode resistance gene *Gro1*-*4* by the candidate gene approach (Paal et al. 2004). *R1*, the first gene for resistance to *Phytophthora infestans* causing late blight, the most devastating disease of potato, was cloned by a combination of positional cloning and candidate gene approach based on RGL’s (Ballvora et al. 2002). The molecular mapping of major loci conferring resistance to *Potato Virus Y* (PVY) and the root cyst nematode *Globodera pallida* enabled the development of diagnostic markers for breeding applications (Kasai et al. 2000; Sattarzadeh et al. 2006), reviewed by (Gebhardt 2013). Linkage mapping in tetraploid populations became realistic when large numbers of markers could be generated by labor- and time-effective methods. This was the case when using amplified fragment length polymorphism (AFLP) markers, which could be generated in large numbers without prior cloning or sequencing efforts (Meyer et al. 1998), and, more recently, SNP arrays (Hackett et al. 2014).

Association genetics proved to be a most suitable and fruitful approach for the tetraploid, non-inbred potato, particularly for quantitative agronomic traits. The probably first association test was performed with 383 potato varieties that were evaluated, on the one hand, for field or quantitative resistance to late blight and, on the other hand, for presence of the single *R1* gene for resistance to late blight (identified at that time by infection with avirulent races of *P. infestans* in the presence of *R1*). Cultivars carrying the *R1* gene, which is defeated in the field by *Phytophthora* races with multiple virulence factors, were on average more resistant to late blight than varieties lacking *R1* (Schick et al. 1958). Nearly 50 years later, the feasibility of association mapping with DNA-based markers was tested in a gene bank collection of 415 tetraploid potato cultivars, for which scores for quantitative resistance to late blight were available. Genotyping this population with PCR-based markers within or tightly linked to the now cloned *R1* gene, which was known to co-localize with a major resistance QTL (Leonards-Schippers et al. 1994), revealed that the presence of the *R1* gene was, indeed, associated with increased quantitative resistance to late blight (Gebhardt et al. 2004). In subsequent years, a number of association mapping experiments were performed using various types of DNA-based markers including SNPs, either derived from candidate genes or genome-wide distributed, which discovered further associations with quantitative resistance to late blight and other complex traits such as plant maturity, tuber starch content, yield, starch yield, chip color and enzymatic discoloration, reviewed by (Gebhardt et al. 2014), (Mosquera et al. 2016; Schönhals et al. 2016), see also Bernardo (2016) in this issue.

Roots and tubers of potato (*Solanum tuberosum*), sweet potato (*Ipomoea batatas*), cassava (*Manihot esculenta*) and yam (*Dioscorea* species) are important components of human nutrition. Physiology, biochemistry and genetics of tuber initiation and development were most extensively studied in the potato, beginning more than 100 years ago (Bernard 1901). Tuberization is a complex trait controlled by multiple genetic and environmental factors like photoperiod, temperature and nitrogen supply. Hormones like gibberellins, jasmonates and cytokinins, as well as carbohydrate metabolism play important roles in tuber development, reviewed by (Prat 2010). A number of genes functional in tuberization have been identified, among those were two genes that control the day-length-dependent tuberization. The *StSP6A* gene was cloned by the candidate gene approach. It encodes a protein similar to FLOWERING LOCUS T (FT), which controls day-length-dependent flowering in *Arabidopsis thaliana* (Navarro et al. 2011). The *StCDF1* gene was cloned based on genomic position. It encodes a member of the family of DOF (DNA-binding with one finger) transcription factors (Kloosterman et al. 2013).

## Pepper

The home of the genus *Capsicum* comprising more than thirty species is the tropical America. There, the consumption of *Capsicum* fruits by humans goes back to at least 6000 years (Perry et al. 2007) and their cultivation at least 2500 years. Observing the use of this plant by the indigenous people, the Spanish conquistadores associated it with the black pepper, which was well known and precious to them. This accelerated the introduction and adoption of *Capsicum* species in Europe and Asia from the sixteenth century onwards. Nowadays, hot and sweet peppers are cultivated worldwide and form an important part of the human diet, particularly in Asia. There are five principal cultivated species of hot and sweet pepper: *C. annuum,* the most widely cultivated species, *C. pubescens, C. chinense, C. baccatum and C. frutescens* (Daunay et al. 2008). The earliest study on the Mendelian inheritance of plant architecture, fruit color, shape and flavor of *C. annuum* was conducted by Herbert J. Webber at Cornell University (Webber 1912). One of his findings was that the pungent and sweet flavor was inherited as a single factor with pungent being dominant over sweet. Nearly 100 years later, the gene *Pun1* controlling pungency in pepper was identified as a putative acyltransferase, which catalyzes the last step in the biosynthesis of the alkaloid capsaicin causing the pungent flavor (Stewart et al. 2005). After the discovery of non-Mendelian, cytoplasmic inheritance by Erwin Baur and Carl Correns in 1909 (Hagemann 2010), *C. annum* was the first Solanaceous species, in which cytoplasmic inheritance was observed (Ikeno 1917). Classical genetic analysis remained rather limited in pepper. It mainly dealt with inheritance of characters of the fruit like fruit shape and the yellow, orange, red, green or brown fruit color (Hurtado-Hernandez and Smith 1985; Peterson 1959). The first linkage map of pepper was constructed in progeny of an interspecific cross between *C. annuum* and *C. chinense* using tomato RFLP markers, which cross-hybridized to pepper genomic DNA. The synteny observed between the pepper and tomato maps was used to model the evolution of the two genomes (Prince et al. 1993; Tanksley et al. 1988). In the following 20 years, several molecular maps were built in different genetic backgrounds and with various types of DNA markers. These maps formed the basis for the linkage mapping of numerous qualitative and quantitative horticultural traits such as fruit characters (size, shape, weight, color, carotenoid, anthocyan and capsaicinoid content, pendant/erect fruit habit), disease resistance (resistance to viruses, bacteria, nematodes and the oomycete *Phytophthora capsici*), and male sterility, comprehensively reviewed by (Ramchiary et al. 2014). First results of association mapping of fruit weight and capsaicinoid content have also been obtained (Nimmakayala et al. 2014). Map-based cloning of the *Bs3* gene for resistance to *Xanthomonas campestris* pv. *vesicatoria* revealed that *Bs3* encodes a flavin monooxygenase, a novel type of plant resistance gene (Römer et al. 2007). Other genes controlling important phenotypic characters were identified by the candidate gene approach, for example, the pepper gene orthologous to the tomato *Ovate* gene for fruit shape (Tsaballa et al. 2011) and the recessive gene *pvr1* for resistance to *Potato Virus Y* (PVY) (Ruffel et al. 2002). An important result of molecular mapping and cloning efforts was the development of easy-to-use and cost-effective DNA-based markers that are suitable for breeding applications (Holdsworth and Mazourek 2015; Ramchiary et al. 2014).

An annotated, physically ordered reference genome was assembled from sequencing the Mexican landrace ‘Criollo de Morelos’ of *Capsicum annuum.* The cultivars ‘Perennial’ and ‘Dempsey’ and the species *C. chinense* were re-sequenced and compared with the reference genome and the tomato genome (Kim et al. 2014).

## Eggplant

Eggplant (*Solanum melongena*) is the only crop plant of the Solanaceae family that is indigenous to the Old World, namely to southeast Asia. Eggplant was known in India 2000 years ago and cultivated in China at least 1500 years ago. It was well known in the early Islamic empires in the Middle East and reached Europe probably with the Arab conquest of Spain in the eighth century and Africa about the same time with Arab and Persian traders. Later, it migrated with the Europeans to America (Daunay et al. 2008). Despite its importance as one of the world’s top five vegetable species, genetic research on eggplant did not play a prominent role in the twentieth century. Byron D. Halsted compared the inheritance of the fruit colors of tomato, eggplant and pepper and noticed genetic similarities between them, which might be considered as the first observation of synteny (Halsted 1918). Later, the genetics of fruit and flower colors was refined (Tatebe 1939; Tigchelaar et al. 1968) and the inheritance of male sterility was analyzed, which is important for hybrid breeding (Nuttall 1963). Eggplant was the last to enter the genomics era after tomato, potato and pepper. A linkage map was constructed in F2 progeny of an interspecific hybrid between *S. melongena* and the wild relative *S. linnaeanum* based on 233 tomato RFLP markers (Doganlar et al. 2002a). Recently, an intraspecific map based on 415 SNP and SSR (simple sequence repeat) markers was published (Barchi et al. 2009). The linkage maps were the prerequisite for QTL mapping of morphological and biochemical fruit characters including yield parameters and plant prickliness (Doganlar et al. 2002b; Portis et al. 2014; Toppino et al. 2008). The eggplant fruit is rich in chlorogenic acid, a polyphenol with high nutraceutical potential. An interspecific map between *S. melongena* and *S. incanum* was instrumental for mapping the genes functional in the chlorogenic acid biosynthesis pathway as well as for integrating tomato and other eggplant maps using common markers (Gramazio et al. 2014). First association mapping experiments have also been performed, which identified associations of SSR and SNP markers with a number of fruit and plant characters. QTLs that have been detected previously by linkage mapping were confirmed, and novel QTL were also found (Cericola et al. 2014; Ge et al. 2013; Portis et al. 2015). A draft genome sequence of the typical Asian eggplant cultivar ‘Nakate-Shinkuro’ was published recently (Hirakawa et al. 2014).

## Conclusions and perspectives

During the first two decades of the twentieth century, when the new-borne scientific discipline of genetics began to unfold, tomato, potato, pepper, petunia and eggplant were among the first plant species subjected to Mendelian studies. One of these five, the tomato, gained and maintained in the following six decades the position of a major plant model in classical genetics. Tobacco was the principal model in plant somatic cell genetics, which culminated between 1975 and 1985 in the *Agrobacterium tumefaciens*-mediated, stable transformation of tobacco cells with foreign genes and the regeneration of fertile transgenic plants. This was one aspect of the revolution in plant genetics, which took place in the last two decades of the twentieth century. Another, equally important aspect was the introduction of natural DNA variants as Mendelian markers, which opened up new approaches for the genetic dissection of quantitative and qualitative phenotypic characters, the positional cloning of so-far intractable genes and, as a consequence of this research, for more efficient and precise plant breeding methods based on marker-assisted selection. The tomato had here a pioneering role. Particularly fruitful was the adoption of population genetics in the beginning of the twenty-first century, which greatly facilitates the translation of basic genetic research in practical breeding applications, especially in polyploid and outcrossing crop species. The tetraploid, heterozygous potato was the leading Solanaceous species in this area of genetic research. The first decade of the twenty-first century saw yet another revolution in genetics: based on next-generation sequencing technologies, the sequencing of whole plant genomes became possible, affordable and increasingly routine. Annotated, physically ordered reference genomes of potato, tomato and hot pepper are now available (Kim et al. 2014; PGSC 2011; Sharma et al. 2013; The Tomato Genome Consortium 2012), draft genome sequences of tobacco and eggplant were recently published (Bombarely et al. 2012; Hirakawa et al. 2014; Sierro et al. 2014) and a petunia genome sequence is under construction (Vandenbussche et al. 2016). The majority of the gene repertoire of these plants is also known thanks to large-scale cDNA sequencing efforts and bioinformatic tools for gene annotation based on sequence homology as well as *de novo*. The annotated, physically ordered genome sequence is the genetic map of ultimate precision, which makes the construction of molecular linkage maps obsolete. The recombination frequency between pairs of loci is replaced by the physical distance expressed in base pairs. Any DNA-based marker, for which minimal sequence information (~20 base pairs) is available, can be mapped to the reference genome sequence in silico by a simple BLAST search of the corresponding genome database. This means that any quantitative or qualitative trait can be anchored to the genome sequence via linked or associated DNA sequence-based markers. This makes possible the integration and comparison of single genes and QTL that were mapped in different genetic backgrounds of the same species or syntenic species like tomato and potato, for example (Mosquera et al. 2016). Genomic sequencing and hybridization of total genomic DNA to genome-wide SNP arrays will be the methods of choice for genotyping populations of individuals with known phenotype, to identify SNPs tightly linked or even identical with the genes that control natural phenotypic variation. This will contribute knowledge of the genes and their natural variants, which underlay complex phenotypic traits in plants. This knowledge is scarce compared with knowledge of the phenotypic effects of knock-out mutant alleles and over- or ectopically expressed genes. The Solanaceae family with its long tradition of genetic research on model as well as non-model species, with its wealth of genetic and genomic resources and with its relevance for human nutrition and well-being, is well positioned for being one of the great players in fundamental and applied plant research in the future.

### Author contribution statement

CG wrote the article.

## References

[CR1] Anderson E (1924). Studies on self-sterility VI. The genetic basis of cross-sterility in Nicotiana. Genetics.

[CR2] Anderson MA, Cornish EC, Mau SL, Williams EG, Hoggart R, Atkinson A, Bonig I, Grego B, Simpson R, Roche PJ, Haley JD, Penschow JD, Niall HD, Tregear GW, Coghlan JP, Crawford RJ, Clarke AE (1986). Cloning of cDNA for a stylar glycoprotein associated with expression of self-incompatibility in *Nicotiana alata*. Nature.

[CR3] Ballvora A, Ercolano MR, Weiss J, Meksem K, Bormann CA, Oberhagemann P, Salamini F, Gebhardt C (2002). The *R1* gene for potato resistance to late blight (*Phytophthora infestans*) belongs to the leucine zipper/NBS/LRR class of plant resistance genes. Plant J.

[CR4] Barchi L, Lefebvre V, Sage-Palloix A-M, Lanteri S, Palloix A (2009). QTL analysis of plant development and fruit traits in pepper and performance of selective phenotyping. Theor Appl Genet.

[CR5] Barone A, Ritter E, Schachtschabel U, Debener T, Salamini F, Gebhardt C (1990). Localization by restriction-fragment-length-polymorphism mapping in potato of a major dominant gene conferring resistance to the potato cyst nematode *Globodera rostochiensis*. Mol Gen Genet.

[CR6] Beadle GW, Tatum EL (1941). Genetic control of biochemical reactions in Neurospora. Proc Natl Acad Sci USA.

[CR7] Bendahmane A, Kanyuka K, Baulcombe DC (1999). The *Rx* gene from potato controls separate virus resistance and cell death responses. Plant Cell.

[CR8] Bent A, Kunkel B, Dahlbeck D, Brown K, Schmidt R, Giraudat J, Leung J, Staskawicz B (1994). *RPS2* of *Arabidopsis thaliana*: a leucine-rich repeat class of plant disease resistance genes. Science.

[CR9] Bernard N (1901) Etudes sur la tuberisation. Biology, Faculty of Sciences. University of Paris

[CR10] Bernardo R (2016) Bandwagons I, too, have known. Theor Appl Genet (**this issue**) 10.1007/s00122-016-2772-5 27681088

[CR11] Bernatzky R, Tanksley SD (1986). Toward a saturated linkage map in tomato based on isozymes and random cDNA sequences. Genetics.

[CR12] Bianchi F, Cornelissen PTJ, Gerats AGM, Hogervorst JMW (1978). Regulation of gene action in *Petunia hybrida*: Unstable alleles of a gene for flower colour. Theor Appl Genet.

[CR13] Bindler G, Hoeven R, Gunduz I, Plieske J, Ganal M, Rossi L, Gadani F, Donini P (2006). A microsatellite marker based linkage map of tobacco. Theor Appl Genet.

[CR14] Bindler G, Plieske J, Bakaher N, Gunduz I, Ivanov N, Van der Hoeven R, Ganal M, Donini P (2011). A high density genetic map of tobacco (*Nicotiana tabacum* L.) obtained from large scale microsatellite marker development. Theor Appl Genet.

[CR15] Bolger A, Scossa F, Bolger ME, Lanz C, Maumus F, Tohge T, Quesneville H, Alseekh S, Sorensen I, Lichtenstein G, Fich EA, Conte M, Keller H, Schneeberger K, Schwacke R, Ofner I, Vrebalov J, Xu Y, Osorio S, Aflitos SA, Schijlen E, Jimenez-Gomez JM, Ryngajllo M, Kimura S, Kumar R, Koenig D, Headland LR, Maloof JN, Sinha N, van Ham RCHJ, Lankhorst RK, Mao L, Vogel A, Arsova B, Panstruga R, Fei Z, Rose JKC, Zamir D, Carrari F, Giovannoni JJ, Weigel D, Usadel B, Fernie AR (2014). The genome of the stress-tolerant wild tomato species *Solanum pennellii*. Nat Genet.

[CR16] Bombarely A, Rosli HG, Vrebalov J, Moffett P, Mueller LA, Martin GB (2012). A draft genome sequence of *Nicotiana benthamiana* to enhance molecular plant-microbe biology research. Mol Plant Microbe Interact.

[CR17] Bonierbale MW, Plaisted RL, Tanksley SD (1988). RFLP maps based on a common set of clones reveal modes of chromosomal evolution in potato and tomato. Genetics.

[CR18] Bonierbale MW, Plaisted RL, Pineda O, Tanksley SD (1994). QTL analysis of trichome-mediated insect resistance in potato. Theor Appl Genet.

[CR19] Bossolini E, Klahre U, Brandenburg A, Reinhardt D, Kuhlemeier C (2011). High resolution linkage maps of the model organism Petunia reveal substantial synteny decay with the related genome of tomato. Genome.

[CR20] Bourgin JP, Nitsch JP (1967). Obtention de Nicotiana haploïdes à partir d’étamines cultivées in vitro (production of haploid nicotiana from excised stamen). Ann Physio Veg.

[CR21] Busch BL, Schmitz G, Rossmann S, Piron F, Ding J, Bendahmane A, Theres K (2011). Shoot branching and leaf dissection in tomato are regulated by homologous gene modules. Plant Cell.

[CR22] Cadman CH (1942). Autotetraploid-inheritance in the potato: some new evidence. J Genet.

[CR23] Carlson PS (1970). Induction and isolation of auxotrophic mutants in somatic cell cultures of Nicotiana tabacum. Science.

[CR24] Carlson PS (1973). Methionine sulfoximine-resistant mutants of tobacco. Science.

[CR25] Cericola F, Portis E, Lanteri S, Toppino L, Barchi L, Acciarri N, Pulcini L, Sala T, Rotino GL (2014). Linkage disequilibrium and genome-wide association analysis for anthocyanin pigmentation and fruit color in eggplant. BMC Genom.

[CR26] Chilton M-D, Drummond MH, Merlo DJ, Sciaky D, Montoya AL, Gordon MP, Nester EW (1977). Stable incorporation of plasmid DNA into higher plant cells: the molecular basis of crown gall tumorigenesis. Cell.

[CR27] Cockerham G (1970). Genetical studies on resistance to potato viruses X and Y. Heredity.

[CR28] Craig AG (1907). Mendel’s law applied in tomato breeding. Proc Soc Hortic Sci.

[CR29] Daunay M-C, Laterrot H, Janick J (2008) Iconography and History of Solanaceae: Antiquity to the 17th Century. Horticultural Reviews. Wiley, New York, pp 1–111

[CR30] De Block M, Herrera-Estrella L, Van Montagu M, Schell J, Zambryski P (1984). Expression of foreign genes in regenerated plants and in their progeny. EMBO J.

[CR31] de Vlaming P, Gerats AGM, Wiering H, Wijsman HJW, Cornu A, Farcy E, Maizonnier D (1984). Petunia hybrida: a short description of the action of 91 genes, their origin and their map location. Plant Mol Biol Report.

[CR32] Doganlar S, Frary A, Daunay M-C, Lester RN, Tanksley SD (2002). A comparative genetic linkage map of eggplant (*Solanum melongena*) and its implications for genome evolution in the Solanaceae. Genetics.

[CR33] Doganlar S, Frary A, Daunay M-C, Lester RN, Tanksley SD (2002). Conservation of gene function in the Solanaceae as revealed by comparative mapping of domestication traits in eggplant. Genetics.

[CR34] Donson J, Kearney CM, Hilf ME, Dawson WO (1991). Systemic expression of a bacterial gene by a tobacco mosaic virus-based vector. Proc Natl Acad Sci USA.

[CR35] Doodeman M, Boersma EA, Koomen W, Bianchi F (1984). Genetic analysis of instability in *Petunia hybrida*. Theor Appl Genet.

[CR36] Dooner HK, Robbins TP, Jorgensen RA (1991). Genetic and developmental control of anthocyanin biosynthesis. Annu Rev Genet.

[CR37] Douches DS, Freyre R (1994). Identification of genetic factors influencing chip color in diploid potato (*Solanum* spp.). Am Potato J.

[CR38] Dunwell JM, Sunderland N (1973). Anther culture of *Solanum tuberosum* L. Euphytica.

[CR39] East EM, Mangelsdorf AJ (1925). A new interpretation of the hereditary behaviour of self-sterile plants. Proc Natl Acad Sci.

[CR40] East EM, Park JB (1917). Studies on self-sterility I. The behaviour of self-sterile plants. Genetics.

[CR41] Eshed Y, Zamir D (1995). An introgression line population of *Lycopersicon pennellii* in the cultivated tomato enables the identification and fine mapping of yield-associated QTL. Genetics.

[CR42] Farcy E, Cornu A (1979). Isolation and characterization of anthocyanin variants originating from the unstable system an2-1 in *Petunia hybrida* (Hort.). Theor Appl Genet.

[CR43] Foolad MR, Panthee DR (2012). Marker-assisted selection in tomato breeding. Crit Rev Plant Sci.

[CR44] Frary A, Nesbitt TC, Frary A, Grandillo S, Evd Knaap, Cong B, Liu J, Meller J, Elber R, Alpert KB, Tanksley SD (2000). fw2.2: a quantitative trait locus key to the evolution of tomato fruit size. Science.

[CR45] Freyre R, Warnke S, Sosinski B, Douches DS (1994). Quantitative trait locus analysis of tuber dormancy in diploid potato (*Solanum* spp.). Theor Appl Genet.

[CR46] Fridman E, Carrari F, Liu Y-S, Fernie AR, Zamir D (2004). Zooming in on a quantitative trait for tomato yield using interspecific introgressions. Science.

[CR47] Ge HY, Liu Y, Zhang J, Han HQ, Li HZ, Shao WT, Chen HY (2013). Simple sequence repeat-based association analysis of fruit traits in eggplant (*Solanum melongena*). Genet Mol Res.

[CR48] Gebhardt C (2013). Bridging the gap between genome analysis and precision breeding in potato. Trends Genet.

[CR49] Gebhardt C, Schnebli V, King PJ (1981). Isolation of biochemical mutants using haploid mesophyll protoplasts of *Hyoscyamus muticus*. Planta.

[CR50] Gebhardt C, Ritter E, Debener T, Schachtschabel U, Walkemeier B, Uhrig H, Salamini F (1989). RFLP analysis and linkage mapping in *Solanum tuberosum*. Theor Appl Genet.

[CR51] Gebhardt C, Ritter E, Barone A, Debener T, Walkemeier B, Schachtschabel U, Kaufmann H, Thompson RD, Bonierbale MW, Ganal MW, Tanksley SD, Salamini F (1991). RFLP maps of potato and their alignment with the homoeologous tomato genome. Theor Appl Genet.

[CR52] Gebhardt C, Walkemeier B, Henselewski H, Barakat A, Delseny M, Stuber K (2003). Comparative mapping between potato (*Solanum tuberosum*) and *Arabidopsis thaliana* reveals structurally conserved domains and ancient duplications in the potato genome. Plant J.

[CR53] Gebhardt C, Ballvora A, Walkemeier B, Oberhagemann P, Schüler K (2004). Assessing genetic potential in germplasm collections of crop plants by marker-trait association: a case study for potatoes with quantitative variation of resistance to late blight and maturity type. Mol Breed.

[CR54] Gebhardt C, Urbany C, Stich B, Tuberosa R, Graner A, Frison E (2014). Dissection of potato complex traits by linkage and association genetics as basis for developing molecular diagnostics in breeding programs. Genomics of plant genetic resources.

[CR55] Gerats T, Vandenbussche M (2005). A model system for comparative research: *Petunia*. Trends Plant Sci.

[CR56] Gerats AG, Huits H, Vrijlandt E, Maraña C, Souer E, Beld M (1990). Molecular characterization of a nonautonomous transposable element (dTph1) of petunia. Plant Cell.

[CR57] Gerats T, Zethof J, Vandenbussche M, Peterson T (2013). Identification and applications of the petunia class II Act1/dTph1 transposable element system. Plant transposable elements: methods and protocols.

[CR58] Giovannoni JJ (2007). Fruit ripening mutants yield insights into ripening control. Curr Opin Plant Biol.

[CR59] Goodin MM, Zaitlin D, Naidu RA, Lommel SA (2008). *Nicotiana benthamiana*: its history and future as a model for plant–pathogen interactions. Mol Plant Microbe Interact.

[CR60] Gramazio P, Prohens J, Plazas M, Andújar I, Herraiz FJ, Castillo E, Knapp S, Meyer RS, Vilanova S (2014). Location of chlorogenic acid biosynthesis pathway and polyphenol oxidase genes in a new interspecific anchored linkage map of eggplant. BMC Plant Biol.

[CR61] Grandillo S, Tanksley SD (1996). QTL analysis of horticultural traits differentiating the cultivated tomato from the closely related species *Lycopersicon pimpinellifolium*. Theor Appl Genet.

[CR62] Grandillo S, Termolino P, van der Knaap E (2013) Molecular mapping of complex traits in tomato. In: Liedl BE, Labate JA, Stommel JR, Slade A, Kole C (eds) Genetics, Genomics, and Breeding of Tomato. Science Publishers, pp 150–227

[CR63] Guha S, Maheshwari SC (1966). Cell division and differentiation of embryos in the pollen grains of *Datura* in vitro. Nature.

[CR64] Hackett CA, Bradshaw JE, Bryan GJ (2014). QTL mapping in autotetraploids using SNP dosage information. Theor Appl Genet.

[CR65] Hagemann R (2010). The foundation of extranuclear inheritance: plastid and mitochondrial genetics. Mol Genet Genomics.

[CR66] Halsted BD (1905) Report of the Botanist. New Jersey Agricultural Experimental Station:423–525

[CR67] Halsted BD (1918). Colors in vegetable fruits. J Heredity.

[CR68] Hawkes JG (1990). The potato, evolution, biodiversity and genetic resources.

[CR69] He C, Saedler H (2005) Heterotopic expression of *MPF2* is the key to the evolution of the Chinese lantern of *Physalis*, a morphological novelty in Solanaceae. Proc Natl Acad Sci USA 102:5779–5784 10.1073/pnas.0501877102 PMC556287 15824316

[CR70] He C, Saedler H (2007). Hormonal control of the inflated calyx syndrome, a morphological novelty, in *Physalis*. Plant J.

[CR71] Hedrick UP, Booth NO (1907) Mendelian characters in tomatoes. In: Proceedings of the Society for Horticultural Science 5

[CR72] Heijmans K, Ament K, Rijpkema AS, Zethof J, Wolters-Arts M, Gerats T, Vandenbussche M (2012). Redefining C and D in the *Petunia* ABC. Plant Cell.

[CR73] Helentjaris T, King G, Slocum M, Siedenstrang C, Wegman S (1985). Restriction fragment polymorphisms as probes for plant diversity and their development as tools for applied plant breeding. Plant Mol Biol.

[CR74] Helentjaris T, Slocum M, Wright S, Schaefer A, Nienhuis J (1986). Construction of genetic linkage maps in maize and tomato using restriction fragment length polymorphisms. Theor Appl Genet.

[CR75] Hernalsteens J-P, Vliet FV, Beuckeleer MD, Depicker A, Engler G, Lemmers M, Holsters M, Montagu MV, Schell J (1980). The *Agrobacterium tumefaciens* Ti plasmid as a host vector system for introducing foreign DNA in plant cells. Nature.

[CR76] Hirakawa H, Shirasawa K, Miyatake K, Nunome T, Negoro S, Ohyama A, Yamaguchi H, Sato S, Isobe S, Tabata S, Fukuoka H (2014). Draft genome sequence of eggplant (*Solanum melongena* L.): the representative *Solanum* species indigenous to the Old World. DNA Res.

[CR77] Holdsworth WL, Mazourek M (2015). Development of user-friendly markers for the pvr1 and Bs3 disease resistance genes in pepper. Mol Breeding.

[CR78] Holmes FO (1938). Inheritance of resistance to tobacco-mosaic disease in tobacco. Phytopathology.

[CR79] Holton TA, Cornish EC (1995). Genetics and biochemistry of anthocyanin biosynthesis. Plant Cell.

[CR80] Horsch RB, Fraley RT, Rogers SG, Sanders PR, Lloyd A, Hoffmann N (1984). Inheritance of functional foreign genes in plants. Science.

[CR81] Horsch RB, Fry JE, Hoffmann NL, Eichholtz D, Rogers SG, Fraley RT (1985). A simple and general method for transferring genes into plants. Science.

[CR82] Hosaka K, Hanneman ER (1998). Genetics of self-compatibility in a self-incompatible wild diploid potato species *Solanum chacoense*. 2. Localization of an *S* locus inhibitor (*Sli*) gene on the potato genome using DNA markers. Euphytica.

[CR83] Hougas RW, Peloquin SJ, Ross RW (1958). Haploids of the common potato. J Hered.

[CR84] Hurtado-Hernandez H, Smith PG (1985). Inheritance of mature fruit color in *Capsicum annuum* L. J Hered.

[CR85] Ikeno S (1917). Studies on the hybrids of *Capsicum annuum*. Part II. On some variegated races. J Genet.

[CR86] Jones DF (1917). Linkage in *lycopersicum*. Am Nat.

[CR87] Kasai K, Morikawa Y, Sorri VA, Valkonen JP, Gebhardt C, Watanabe KN (2000). Development of SCAR markers to the PVY resistance gene *Ry*_*adg*_ based on a common feature of plant disease resistance genes. Genome.

[CR88] Kim S, Park M, Yeom S-I, Kim Y-M, Lee JM, Lee H-A, Seo E, Choi J, Cheong K, Kim K-T, Jung K, Lee G-W, Oh S-K, Bae C, Kim S-B, Lee H-Y, Kim S-Y, Kim M-S, Kang B-C, Jo YD, Yang H-B, Jeong H-J, Kang W-H, Kwon J-K, Shin C, Lim JY, Park JH, Huh JH, Kim J-S, Kim B-D, Cohen O, Paran I, Suh MC, Lee SB, Kim Y-K, Shin Y, Noh S-J, Park J, Seo YS, Kwon S-Y, Kim HA, Park JM, Kim H-J, Choi S-B, Bosland PW, Reeves G, Jo S-H, Lee B-W, Cho H-T, Choi H-S, Lee M-S, Yu Y, Do Choi Y, Park B-S, van Deynze A, Ashrafi H, Hill T, Kim WT, Pai H-S, Ahn HK, Yeam I, Giovannoni JJ, Rose JKC, Sorensen I, Lee S-J, Kim RW, Choi I-Y, Choi B-S, Lim J-S, Lee Y-H, Choi D (2014). Genome sequence of the hot pepper provides insights into the evolution of pungency in *Capsicum* species. Nat Genet.

[CR89] Klee HJ, Giovannoni JJ (2011). Genetics and control of tomato fruit ripening and quality attributes. Annu Rev Genet.

[CR90] Kloosterman B, Abelenda JA, Gomez MdMC, Oortwijn M, de Boer JM, Kowitwanich K, Horvath BM, van Eck HJ, Smaczniak C, Prat S, Visser RGF, Bachem CWB (2013). Naturally occurring allele diversity allows potato cultivation in northern latitudes. Nature.

[CR91] Knapp S, Bohs L, Nee M, Spooner DM (2004). Solanaceae—a model for linking genomics with biodiversity. Comp Funct Genom.

[CR92] Kramer MG, Redenbaugh K (1994). Commercialization of a tomato with an antisense polygalacturonase gene: the FLAVR SAVR™ tomato story. Euphytica.

[CR93] Krausch H-D (2007) “Kaiserkron und Päonien rot….” Von der Entdeckung und Einführung unserer Gartenblumen. Deutscher Taschenbuch Verlag München

[CR94] Kumagai MH, Donson J, della-Cioppa G, Harvey D, Hanley K, Grill LK (1995). Cytoplasmic inhibition of carotenoid biosynthesis with virus-derived RNA. Proc Natl Acad Sci USA.

[CR95] Leister D, Ballvora A, Salamini F, Gebhardt C (1996). A PCR-based approach for isolating pathogen resistance genes from potato with potential for wide application in plants. Nat Genet.

[CR96] Leonards-Schippers C, Gieffers W, Salamini F, Gebhardt C (1992). The *R1* gene conferring race-specific resistance to *Phytophthora infestans* in potato is located on potato chromosome V. Mol Gen Genet.

[CR97] Leonards-Schippers C, Gieffers W, Schafer-Pregl R, Ritter E, Knapp SJ, Salamini F, Gebhardt C (1994). Quantitative resistance to Phytophthora infestans in potato: a case study for QTL mapping in an allogamous plant species. Genetics.

[CR98] Lindhout P, Meijer D, Schotte T, Hutten RCB, Visser RGF, van Eck HJ (2011). Towards F1 hybrid seed potato breeding. Potato Res.

[CR99] Liu J, Van Eck J, Cong B, Tanksley SD (2002). A new class of regulatory genes underlying the cause of pear-shaped tomato fruit. Proc Natl Acad Sci USA.

[CR100] Lunden AP (1960). Some more evidence of autotetraploid inheritance in the potato (*Solanum tuberosum*). Euphytica.

[CR101] Mach J (2012). A petunia twist on the ABC model of floral organ specification. Plant Cell.

[CR102] Martin G, Brommonschenkel S, Chunwongse J, Frary A, Ganal M, Spivey R, Wu T, Earle E, Tanksley S (1993). Map-based cloning of a protein kinase gene conferring disease resistance in tomato. Science.

[CR103] Martin GB, de Vicente CM, Tanksley SD (1993). High-resolution linkage analysis and physical characterization of the *Pto* bacterial resistance locus in tomato. Mol Plant Microbe Interact.

[CR104] Márton L, Dung TM, Mendel RR, Maliga P (1982). Nitrate reductase deficient cell lines from haploid protoplast cultures of *Nicotiana plumbaginifolia*. Mol Gen Genet.

[CR105] Márton L, Sidorov V, Biasini G, Maliga P (1982). Complementation in somatic hybrids indicates four types of nitrate reductase deficient lines in Nicotiana plumbaginifolia. Mol Gen Genet.

[CR106] Mather K, Edwardes PMJ (1943). Specific differences in *Petunia* III. Flower color and genetic isolation. J Genet.

[CR107] McClure BA, Haring V, Ebert PR, Anderson MA, Simpson RJ, Sakiyama F, Clarke AE (1989). Style self-incompatibility gene products of Nicotiana alata are ribonucleases. Nature.

[CR108] McClure BA, Gray JE, Anderson MA, Clarke AE (1990). Self-incompatibility in *Nicotiana alata* involves degradation of pollen rRNA. Nature.

[CR109] Menda N, Semel Y, Peled D, Eshed Y, Zamir D (2004). In silico screening of a saturated mutation library of tomato. Plant J.

[CR110] Mendel G (1866). Versuche über Pflanzen-Hybriden. Verhandlungen des Naturforschenden Vereins zu Brünn.

[CR111] Meyer P, Heidmann I, Forkmann G, Saedler H (1987). A new petunia flower colour generated by transformation of a mutant with a maize gene. Nature.

[CR112] Meyer P, Linn F, Heidmann I, Meyer H, Niedenhof I, Saedler H (1992). Endogenous and environmental factors influence 35S promoter methylation of a maize A1 gene construct in transgenic petunia and its colour phenotype. Mol Gen Genet.

[CR113] Meyer RC, Milbourne D, Hackett CA, Bradshaw JE, McNichol JW, Waugh R (1998). Linkage analysis in tetraploid potato and association of markers with quantitative resistance to late blight (*Phytophthora infestans*). Mol Gen Genet.

[CR114] Mindrinos M, Katagiri F, Yu G-L, Ausubel FM (1994). The *A. thaliana* disease resistance gene *RPS2* encodes a protein containing a nucleotide-binding site and leucine-rich repeats. Cell.

[CR115] Mosquera T, Alvarez MF, Jiménez-Gómez JM, Muktar MS, Paulo MJ, Steinemann S, Li J, Draffehn A, Hofmann A, Lübeck J, Strahwald J, Tacke E, Hofferbert H-R, Walkemeier B, Gebhardt C (2016). Targeted and untargeted approaches unravel novel candidate genes and diagnostic SNPs for quantitative resistance of the potato (*Solanum tuberosum* L.) to *Phytophthora infestans* causing the late blight disease. PLoS One.

[CR116] Muir WH, Hildebrandt AC, Riker AJ (1954). Plant tissue cultures produced from single isolated cells. Science.

[CR117] Müller AJ, Grafe R (1978). Isolation and characterization of cell lines of *Nicotiana tabacum* lacking nitrate reductase. Mol Gen Genet.

[CR118] Murashige T, Skoog F (1962). A revised medium for rapid growth and bio assays with tobacco tissue cultures. Physiol Plant.

[CR119] Mutschler MA, Tanksley SD, Rick CM (1987). 1987 Linkage maps of the tomato (*Lycopersicum esculentum*). TGC Report.

[CR120] Nagata T, Takebe I (1971). Plating of isolated tobacco mesophyll protoplasts on agar medium. Planta.

[CR121] Nakata K, Tanaka M (1968). Differentiation of embryoids from developing germ cells in anther culture of tobacco. Jpn J Genet.

[CR122] Napoli C, Lemieux C, Jorgensen R (1990). Introduction of a chimeric chalcone synthase gene into petunia results in reversible co-suppression of homologous genes in trans. Plant Cell.

[CR123] Navarro C, Abelenda JA, Cruz-Oro E, Cuellar CA, Tamaki S, Silva J, Shimamoto K, Prat S (2011). Control of flowering and storage organ formation in potato by FLOWERING LOCUS T. Nature.

[CR124] Negrutiu I, Jacobs M, Caboche M (1984). Advances in somatic cell genetics of higher plants—the protoplast approach in basic studies on mutagenesis and isolation of biochemical mutants. Theor Appl Genet.

[CR125] Nienhuis J, Helentjaris T, Slocum M, Ruggero B, Schaefer A (1987). Restriction fragment length polymorphism analysis of loci associated with insect resistance in tomato. Crop Sci.

[CR126] Nimmakayala P, Abburi VL, Abburi L, Alaparthi SB, Cantrell R, Park M, Choi D, Hankins G, Malkaram S, Reddy UK (2014). Linkage disequilibrium and population-structure analysis among *Capsicum annuum* L. cultivars for use in association mapping. Mol Genet Genom.

[CR127] Nitsch JP, Nitsch C (1969). Haploid plants from pollen grains. Science.

[CR128] Nuttall VW (1963). The inheritance and possible usefulness of functional male sterility in *Solanum melongena* L.. Can J Genet Cytol.

[CR129] O’Reilly C, Shepherd NS, Pereira A, Schwarz-Sommer Z, Bertram I, Robertson DS, Peterson PA, Saedler H (1985). Molecular cloning of the *a1* locus of *Zea mays* using the transposable elements En and Mu1. The EMBO J.

[CR130] Osborn TC, Alexander DC, Fobes JF (1987). Identification of restriction fragment length polymorphisms linked to genes controlling soluble solids content in tomato fruit. Theor Appl Genet.

[CR131] Paal J, Henselewski H, Muth J, Meksem K, Menendez CM, Salamini F, Ballvora A, Gebhardt C (2004). Molecular cloning of the potato *Gro1*-*4* gene conferring resistance to pathotype Ro1 of the root cyst nematode *Globodera rostochiensis*, based on a candidate gene approach. Plant J.

[CR132] Pascual L, Desplat N, Huang BE, Desgroux A, Bruguier L, Bouchet J-P, Le QH, Chauchard B, Verschave P, Causse M (2015). Potential of a tomato MAGIC population to decipher the genetic control of quantitative traits and detect causal variants in the resequencing era. Plant Biotechnol J.

[CR133] Paterson AH, Lander ES, Hewitt JD, Peterson S, Lincoln SE, Tanksley SD (1988). Resolution of quantitative traits into Mendelian factors by using a complete linkage map of restriction fragment length polymorphisms. Nature.

[CR134] Perry L, Dickau R, Zarrillo S, Holst I, Pearsall DM, Piperno DR, Berman MJ, Cooke RG, Rademaker K, Ranere AJ, Raymond JS, Sandweiss DH, Scaramelli F, Tarble K, Zeidler JA (2007). Starch fossils and the domestication and dispersal of chili peppers (*Capsicum* spp. L.) in the Americas. Science.

[CR135] Peterson PA (1959). Linkage of fruit shape and color genes in *Capsicum*. Genetics.

[CR136] PGSC (2011). Genome sequence and analysis of the tuber crop potato. Nature.

[CR137] Portis E, Barchi L, Toppino L, Lanteri S, Acciarri N, Felicioni N, Fusari F, Barbierato V, Cericola F, Valè G, Rotino GL (2014). QTL mapping in eggplant reveals clusters of yield-related loci and orthology with the tomato genome. PLoS One.

[CR138] Portis E, Cericola F, Barchi L, Toppino L, Acciarri N, Pulcini L, Sala T, Lanteri S, Rotino GL (2015). Association mapping for fruit, plant and leaf morphology traits in eggplant. PLoS One.

[CR139] Prat S, Davies PJ (2010). Hormonal and daylength control of potato tuberization. Plant hormones: biosynthesis, signal transduction, action!.

[CR140] Price HL, Drinkard AW (1908). Inheritance in tomato hybrids. Va Agric Exp Sta Bull.

[CR141] Prince JP, Pochard E, Tanksley SD (1993). Construction of a molecular linkage map of pepper and a comparison of synteny with tomato. Genome.

[CR142] Ramchiary N, Kehie M, Brahma V, Kumaria S, Tandon P (2014). Application of genetics and genomics towards *Capsicum* translational research. Plant Biotechnol Rep.

[CR143] Ranc N, Muños S, Xu J, Le Paslier M-C, Chauveau A, Bounon R, Rolland S, Bouchet J-P, Brunel D, Causse M (2012) Genome-wide association mapping in tomato (*Solanum lycopersicum*) is possible using genome admixture of *Solanum lycopersicum* var. *cerasiforme*. G3: Genes|Genomes|Genetics 2:853–864 10.1534/g3.112.002667 PMC3411241 22908034

[CR144] Reif HJ, Niesbach U, Deumling B, Saedler H (1985). Cloning and analysis of two genes for chalcone synthase from *Petunia hybrida*. Mol Gen Genet.

[CR145] Ríos D, Ghislain M, Rodríguez F, Spooner DM (2007). What is the origin of the European potato? Evidence from Canary island landraces. Crop Sci.

[CR146] Ritter E, Debener T, Barone A, Salamini F, Gebhardt C (1991). RFLP mapping on potato chromosomes of two genes controlling extreme resistance to potato virus X (PVX). Mol Gen Genet.

[CR147] Rokka V-M, Touraev A, Forster BP, Jain SM (2009). Potato Haploids and Breeding. Advances in haploid production in higher plants.

[CR148] Römer P, Hahn S, Jordan T, Strauß T, Bonas U, Lahaye T (2007). Plant pathogen recognition mediated by promoter activation of the pepper *Bs3* resistance gene. Science.

[CR149] Ruffel S, Dussault M-H, Palloix A, Moury B, Bendahmane A, Robaglia C, Caranta C (2002). A natural recessive resistance gene against potato virus Y in pepper corresponds to the eukaryotic initiation factor 4E (eIF4E). Plant J.

[CR150] Ruggieri V, Francese G, Sacco A, D’Alessandro A, Rigano MM, Parisi M, Milone M, Cardi T, Mennella G, Barone A (2014). An association mapping approach to identify favourable alleles for tomato fruit quality breeding. BMC Plant Biol.

[CR151] Rybin WA (1930). Karyologische Untersuchungen an einigen wilden und einheimischen kultivierten Kartoffeln Amerikas. Zeitschrift für Induktive Abstammungs- und Vererbungslehre.

[CR152] Saito T, Ariizumi T, Okabe Y, Asamizu E, Hiwasa-Tanase K, Fukuda N, Mizoguchi T, Yamazaki Y, Aoki K, Ezura H (2011). TOMATOMA: a novel tomato mutant database distributing micro-tom mutant collections. Plant Cell Physiol.

[CR153] Salaman R (1910). The inheritance of colour and other characters in the potato. J Genet.

[CR154] Sattarzadeh A, Achenbach U, Lübeck J, Strahwald J, Tacke E, Hofferbert HR, Rothsteyn T, Gebhardt C (2006). Single nucleotide polymorphism (SNP) genotyping as basis for developing a PCR-based marker highly diagnostic for potato varieties with high resistance to *Globodera pallida* pathotype *Pa2/3*. Mol Breed.

[CR155] Saunders ER (1910). Studies in the inheritance of doubleness in flowers. J Genet.

[CR156] Sauvage C, Segura V, Bauchet G, Stevens R, Thi Do P, Nikoloski Z, Fernie AR, Causse M (2014). Genome-wide association in tomato reveals 44 candidate loci for fruit metabolic traits. Plant Physiol.

[CR157] Schick R, Möller KH, Haussdörfer M, Schick E (1958). Die Widerstandsfähigkeit von Kartoffelsorten gegenüber der durch *Phytophthora infestans*(Mont.) De Bary hervorgerufenen Krautfäule. Der Züchter.

[CR158] Schmitz G, Tillmann E, Carriero F, Fiore C, Cellini F, Theres K (2002). The tomato *Blind* gene encodes a MYB transcription factor that controls the formation of lateral meristems. Proc Natl Acad Sci USA.

[CR159] Scholthof K-BG (2008) Tobacco mosaic virus: the beginning of plant pathology. APSnet Features

[CR160] Schönhals EM, Ortega F, Barandalla L, Aragones A, Ruiz de Galarreta JI, Liao J-C, Sanetomo R, Walkemeier B, Tacke E, Ritter E, Gebhardt C (2016). Identification and reproducibility of diagnostic DNA markers for tuber starch and yield optimization in a novel association mapping population of potato (*Solanum tuberosum* L.). Theor Appl Genet.

[CR161] Schumacher K, Schmitt T, Rossberg M, Schmitz G, Theres K (1999). The *Lateral suppressor* (*Ls*) gene of tomato encodes a new member of the VHIID protein family. Proc Natl Acad Sci USA.

[CR162] Schuman MC, Baldwin IT (2016). The layers of plant responses to insect herbivores. Annu Rev Entomol.

[CR163] Schwarz-Sommer Z, Shepherd N, Tacke E, Gierl A, Rohde W, Leclercq L, Mattes M, Berndtgen R, Peterson PA, Saedler H (1987). Influence of transposable elements on the structure and function of the *Al* gene of *Zea mays*. EMBO J.

[CR164] Schwarz-Sommer Z, Huijser P, Nacken W, Saedler H, Sommer H (1990). Genetic control of flower development by homeotic genes in *Antirrhinum majus*. Science.

[CR165] Sharma SK, Bolser D, de Boer J, Sønderkær M, Amoros W, Carboni MF, D’Ambrosio JM, de la Cruz G, Di Genova A, Douches DS, Eguiluz M, Guo X, Guzman F, Hackett CA, Hamilton JP, Li G, Li Y, Lozano R, Maass A, Marshall D, Martinez D, McLean K, Mejía N, Milne L, Munive S, Nagy I, Ponce O, Ramirez M, Simon R, Thomson SJ, Torres Y, Waugh R, Zhang Z, Huang S, Visser RGF, Bachem CWB, Sagredo B, Feingold SE, Orjeda G, Veilleux RE, Bonierbale M, Jacobs JME, Milbourne D, Martin DMA, Bryan GJ (2013) Construction of reference chromosome-scale pseudomolecules for potato: Integrating the potato genome with genetic and physical maps. G3: Genes|Genom|Genet 3:2031–2047 10.1534/g3.113.007153 PMC3815063 24062527

[CR166] Shirasawa K, Isobe S, Hirakawa H, Asamizu E, Fukuoka H, Just D, Rothan C, Sasamoto S, Fujishiro T, Kishida Y, Kohara M, Tsuruoka H, Wada T, Nakamura Y, Sato S, Tabata S (2010). SNP discovery and linkage map construction in cultivated tomato. DNA Res: Int J Rapid Publ Reports on Genes and Genom.

[CR167] Sierro N, Battey JND, Ouadi S, Bakaher N, Bovet L, Willig A, Goepfert S, Peitsch MC, Ivanov NV (2014) The tobacco genome sequence and its comparison with those of tomato and potato. Nat Commun 5:3833. doi:10.1038/ncomms4833 10.1038/ncomms4833 PMC4024737 24807620

[CR168] Simko I, Jansky S, Stephenson S, Spooner D, Bradshaw J, Gebhardt C, Govers F, Mackkerron DKL, Taylor MA, Ross HA (2007). Genetics of resistance to pests and disease. Potato biology and biotechnology.

[CR169] Sink KC (1984). Petunia.

[CR170] Skoog F (1944). Growth and organ formation in tobacco tissue cultures. Am J Bot.

[CR171] Skoog F, Tsui C (1948). Chemical control of growth and bud formation in tobacco stem segments and callus cultured in vitro. Am J Bot.

[CR172] Smith HB (1927). Chromosome counts in the varieties of *Solanum tuberosum* and allied wild species. Genetics.

[CR173] Stewart C, Kang B-C, Liu K, Mazourek M, Moore SL, Yoo EY, Kim B-D, Paran I, Jahn MM (2005). The *Pun1* gene for pungency in pepper encodes a putative acyltransferase. Plant J.

[CR174] Strommer J, Peters J, Zethof J, de Keukeleire P, Gerats T (2002). AFLP maps of *Petunia hybrida*: building maps when markers cluster. Theor Appl Genet.

[CR175] Stubbe H (1957). Mutanten der Kulturtomate *Lycopersicon esculentum* Miller I. Die Kulturpflanze.

[CR176] Stubbe H (1972). Mutanten der Kulturtomate *Lycopersicon esculentum* Miller VI. Die Kulturpflanze.

[CR177] Sussex IM (2008). The scientific roots of modern plant biotechnology. Plant Cell.

[CR178] Tanksley SD, Bernatzky R, Lapitan NL, Prince JP (1988). Conservation of gene repertoire but not gene order in pepper and tomato. Proc Natl Acad Sci USA.

[CR179] Tanksley SD, Ganal MW, Prince JP, de-Vicente MC, Bonierbale MW, Broun P, Fulton TM, Giovannoni JJ, Grandillo S, Martin GB, Messeguer R, Miller JC, Miller L, Paterson AH, Pineda O, Roder MS, Wing RA, Wu W, Young ND (1992). High density molecular linkage maps of the tomato and potato genomes. Genetics.

[CR180] Tatebe T (1939). On inheritance of color in *Solanum melongena* LINN. Jpn J Genet.

[CR181] The Tomato Genome Consortium (2012). The tomato genome sequence provides insights into fleshy fruit evolution. Nature.

[CR182] Tigchelaar EC, Janick J, Erickson HT (1968). The genetics of anthocyanin coloration in eggplant (*Solanum melongena* L.). Genetics.

[CR183] Toppino L, Vale G, Rotino G (2008). Inheritance of *Fusarium* wilt resistance introgressed from *Solanum aethiopicum Gilo* and Aculeatum groups into cultivated eggplant (*S. melongena*) and development of associated PCR-based markers. Mol Breed.

[CR184] Tsaballa A, Pasentsis K, Darzentas N, Tsaftaris AS (2011). Multiple evidence for the role of an Ovate-like gene in determining fruit shape in pepper. BMC Plant Biol.

[CR185] Van der Krol AR, Chua NH (1993). Flower development in petunia. Plant Cell.

[CR186] Van der Krol AR, Mur LA, Beld M, Mol JN, Stuitje AR (1990). Flavonoid genes in petunia: addition of a limited number of gene copies may lead to a suppression of gene expression. Plant Cell.

[CR187] Van Houwelingen A, Souer E, Spelt K, Kloos D, Mol J, Koes R (1998). Analysis of flower pigmentation mutants generated by random transposon mutagenesis in *Petunia hybrida*. Plant J.

[CR188] Vandenbussche M, Chambrier P, Rodrigues Bento S, Morel P (2016). Petunia, Your Next Supermodel?. Front Plant Sci.

[CR189] Vasil V, Hildebrandt AC (1965). Differentiation of tobacco plants from single, isolated cells in microcultures. Science.

[CR190] Vasil V, Hildebrandt AC (1965). Growth and tissue formation from single, isolated tobacco cells in microculture. Science.

[CR191] Vasil IK, Nitsch C (1975). Experimental production of pollen haploids and their uses. Zeitschrift für Pflanzenphysiologie.

[CR192] Webber HJ (1912). Preliminary notes on pepper hybrids. Ann Rep Am Breed Assoc.

[CR193] White PA (1939). Controlled differentiation in a plant tissue culture. Bull Torrey Bot Club.

[CR194] White PR (1939). Potentially unlimited growth of excised plant callus in an artificial nutrient. Am J Bot.

[CR195] Whitham S, Dinesh-Kumar SP, Choi D, Hehl R, Corr C, Baker B (1994). The product of the tobacco mosaic virus resistance gene *N*: similarity to toll and the interleukin-1 receptor. Cell.

[CR196] Winkel-Shirley B (2001). Flavonoid biosynthesis. A colorful model for genetics, biochemistry, cell biology, and biotechnology. Plant Physiol.

